# Metabolic Dysfunction-Associated Steatotic Liver Disease in Childhood: From Disease Heterogeneity to Personalized Care

**DOI:** 10.3390/jpm16090464

**Published:** 2026-09-08

**Authors:** Maria Rogalidou, Christina Kanaka-Gantenbein

**Affiliations:** 1 Division of Paediatric Gastroenterology, Hepatology, Nutrition, First Department of Paediatrics, Medical School, National & Kapodistrian University of Athens, “Agia Sofia” Children’s Hospital, 115 27 Athens, Greece; 2 Division of Endocrinology, Diabetes and Metabolism, First Department of Paediatrics, Medical School, National & Kapodistrian University of Athens, “Agia Sofia” Children’s Hospital, 115 27 Athens, Greece; chriskan@med.uoa.gr

**Keywords:** pediatric MASLD, metabolic dysfunction-associated steatotic liver disease, personalized medicine, biomarkers, gut microbiome, childhood obesity

## Abstract

Metabolic Dysfunction-Associated Steatotic Liver Disease (MASLD) has become the most common chronic liver disease in childhood, paralleling the global increase in pediatric obesity and metabolic dysfunction. Once considered a benign condition, pediatric MASLD is now recognized as a heterogeneous and potentially progressive disease that may advance from simple steatosis to steatohepatitis, fibrosis, and, rarely, cirrhosis, with lifelong hepatic and cardiometabolic consequences. Its pathogenesis is multifactorial, involving insulin resistance, adipose tissue dysfunction, chronic low-grade inflammation, genetic and epigenetic susceptibility, environmental factors, and alterations in the gut microbiome. Most affected children are asymptomatic, and diagnosis is often prompted by elevated liver enzymes or incidental imaging findings. Noninvasive tools, including ultrasonography, elastography, serum biomarkers, and emerging multi-omics approaches, are improving disease detection and risk stratification, although liver biopsy remains the reference standard in selected cases. Lifestyle modification, including dietary optimization, increased physical activity, and gradual weight reduction, remains the cornerstone of management, while pharmacological therapies are still under investigation in pediatric populations. The marked variability in disease susceptibility; progression; and treatment response underscores the need for a personalized medicine approach. Integrating clinical characteristics with genomic, epigenomic, metabolomic, and microbiome data may enable early identification of high-risk children, more accurate prognostic assessment, and individualized preventive and therapeutic strategies. Early detection and multidisciplinary care involving pediatricians, hepatologists, endocrinologists, dietitians, and families may help reduce disease progression and the risk of long-term hepatic and cardiometabolic complications. This review summarizes current evidence on the epidemiology, pathophysiology, clinical presentation, diagnosis, and management of pediatric MASLD, with a particular emphasis on precision diagnostics, biomarker discovery, and personalized therapeutic approaches. It also discusses current challenges and future directions for implementing personalized medicine to improve outcomes and reduce the lifelong burden of pediatric MASLD.

## 1. Introduction

Metabolic dysfunction-associated steatotic liver disease (MASLD) is one of the most common chronic liver diseases in childhood, particularly among children with overweight or obesity. Following an international expert consensus process (Delphi panel), the terminology was revised. Non-alcoholic fatty liver disease (NAFLD) was renamed metabolic dysfunction-associated steatotic liver disease (MASLD), and non-alcoholic steatohepatitis (NASH) was renamed metabolic dysfunction-associated steatohepatitis (MASH). This change was introduced to emphasize that the disease is primarily driven by metabolic dysfunction rather than being defined by the absence of alcohol consumption [1].

**Figure 1:**
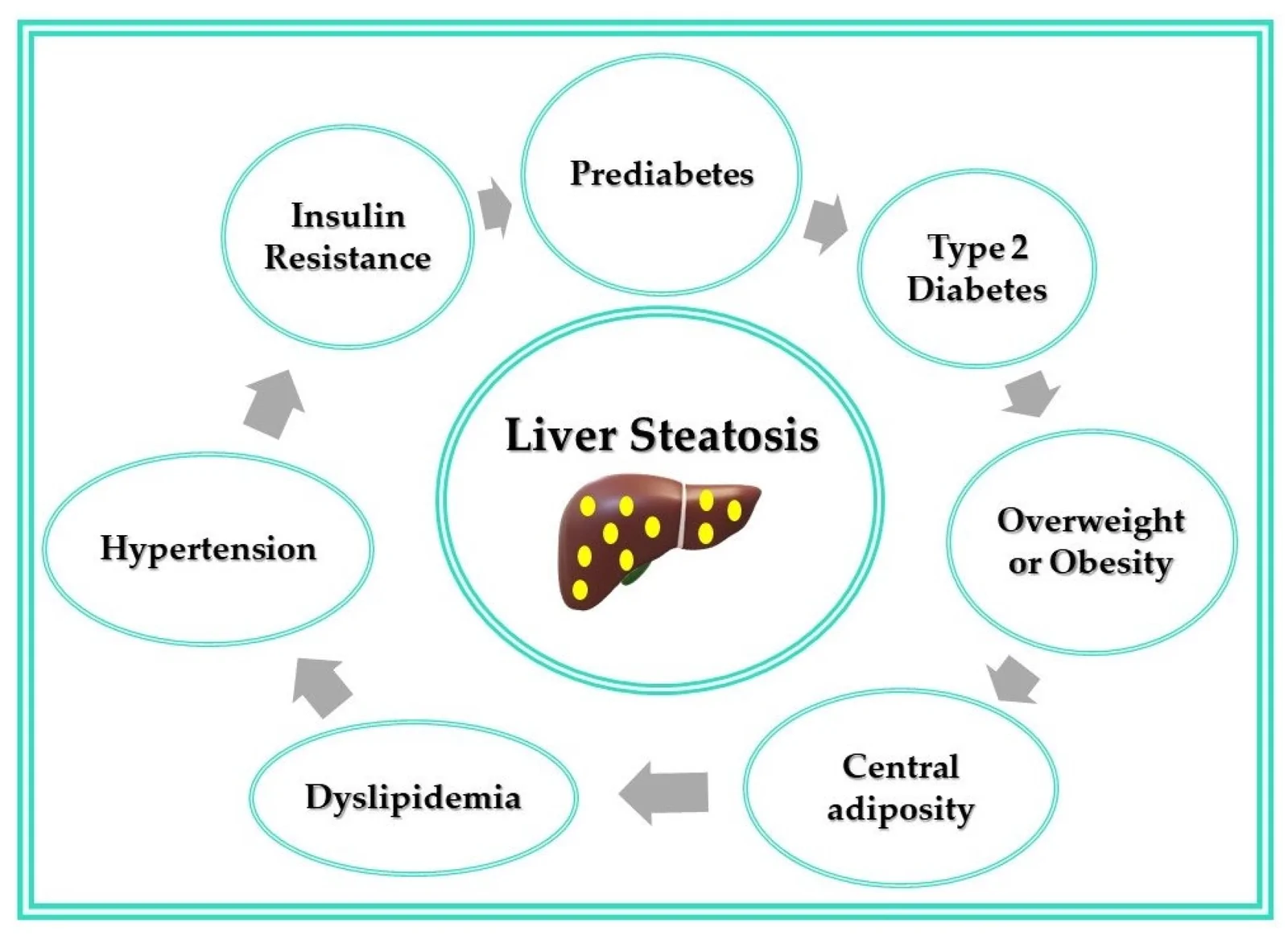
Metabolic dysfunction-associated steatotic liver disease (MASLD) requires evidence of hepatic steatosis in the presence of at least one cardiometabolic risk factor.Yellow shows steatosis.

The diagnosis of metabolic dysfunction-associated steatotic liver disease (MASLD) requires evidence of hepatic steatosis in the presence of at least one cardiometabolic risk factor, such as overweight or obesity, type 2 diabetes mellitus, prediabetes, hypertension, dyslipidemia, insulin resistance, or central obesity as witnessed by increased waist circumference (Figure 1) [1,2,3].

The global epidemic of childhood overweight and obesity has become a major public health concern, affecting children and adolescents worldwide. Large-scale international studies have demonstrated a substantial increase in the prevalence of overweight and obesity over recent decades, with marked differences in disease burden across countries and regions [4,5]. European surveillance studies have similarly reported high and increasing prevalence, with considerable heterogeneity between countries and geographical regions [6,7]. Socioeconomic and demographic factors further contribute to disparities in childhood obesity and its associated comorbidities [8]. This growing burden of childhood obesity is accompanied by an increasing prevalence of metabolic complications, including MASLD, underscoring the importance of early identification of children at risk and the development of individualized strategies for prevention and management. This growing burden of childhood obesity and associated metabolic complications, including MASLD, underscores the importance of early risk identification and individualized approaches to prevention and management.

In addition to excess adiposity, dietary factors play an important role in the development and progression of MASLD. Unhealthy dietary patterns, characterized by excessive intake of energy-dense foods, added sugars, and ultra-processed foods, may contribute to metabolic dysfunction and hepatic steatosis, whereas dietary modification represents an important component of MASLD prevention and management [9]. Prolonged sedentary time and insufficient physical activity have been associated with adverse metabolic profiles and greater hepatic fat accumulation in children and adolescents with obesity [10].

Pediatric MASLD encompasses a broad spectrum of disease, ranging from isolated hepatic steatosis to MASH, a more advanced form characterized by steatosis, inflammation, hepatocellular injury, and variable degrees of fibrosis. Importantly, pediatric MASLD is linked to significant long-term health consequences, including increased mortality and extrahepatic morbidity [11,12].

This review aims to provide a comprehensive overview of the current evidence on the epidemiology, pathophysiology, clinical presentation, diagnosis, and management of pediatric MASLD, with particular emphasis on precision diagnostics, biomarker discovery, and personalized therapeutic approaches. In addition, it examines current challenges and emerging opportunities for the implementation of personalized strategies in the prevention, diagnosis, risk stratification, and management of pediatric MASLD.

### Methods—Literature Search

**Figure 2:**
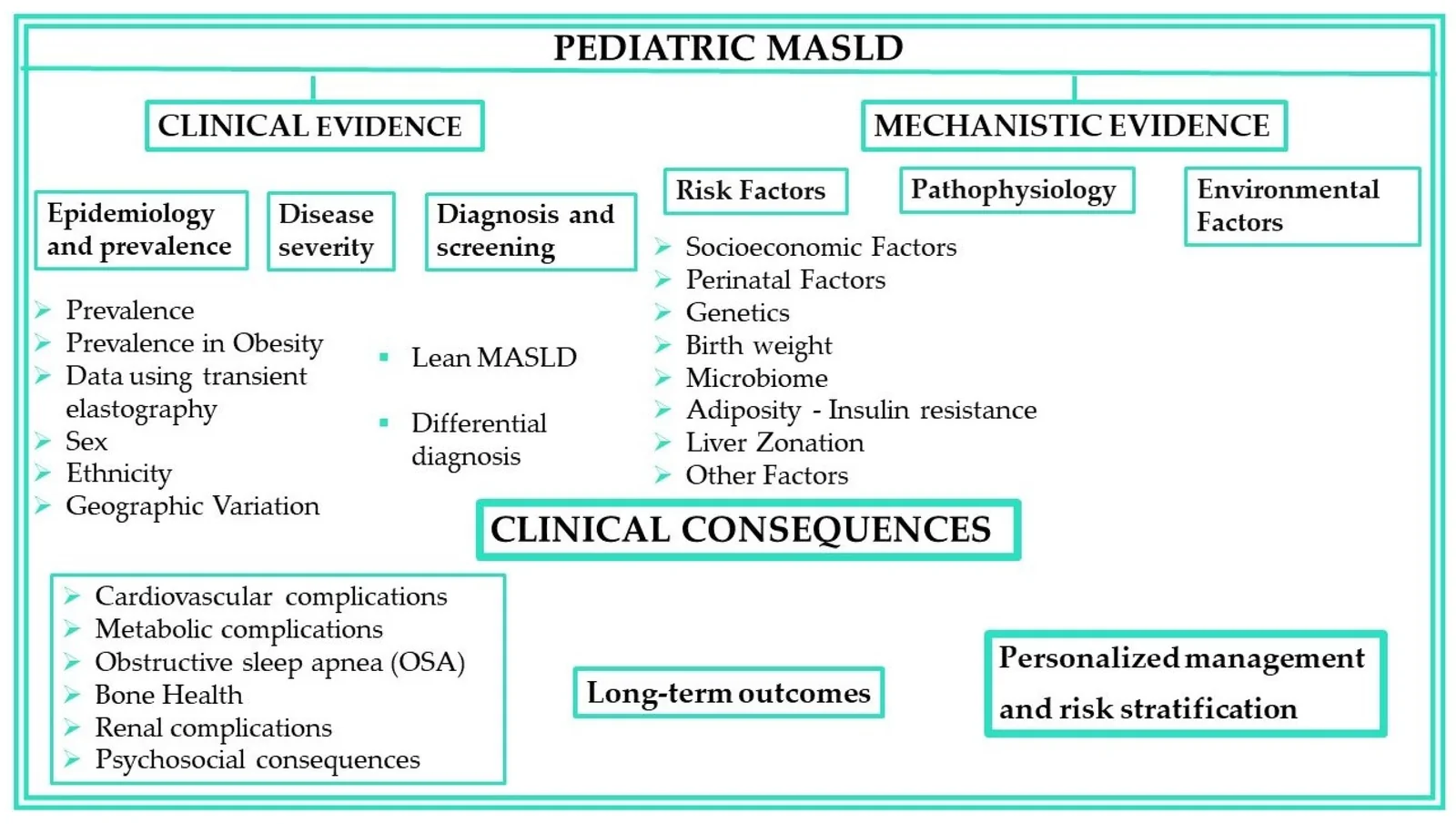
Literature search strategy for pediatric metabolic dysfunction-associated steatotic liver disease (MASLD). Schematic overview of the structured literature search performed to identify relevant clinical and experimental evidence on pediatric MASLD. Searches were organized according to key clinical and mechanistic domains, including epidemiology, diagnosis, pathogenesis, metabolic risk factors, the gut–liver axis, biomarkers, and treatment. Particular emphasis was placed on recent publications, consensus statements, clinical practice guidelines, systematic reviews, meta-analyses, and relevant clinical and experimental studies. Reference lists of key publications were additionally screened to identify eligible and relevant studies.

A structured literature search was performed to identify relevant clinical and experimental evidence concerning pediatric metabolic dysfunction-associated steatotic liver disease (MASLD). The review was organized around key clinical and mechanistic questions, and targeted searches were conducted for each major topic addressed in the manuscript. Relevant publications were identified using appropriate combinations of keywords related to pediatric MASLD, epidemiology, diagnosis, pathogenesis, metabolic risk factors, gut–liver axis, biomarkers, and treatment. Particular emphasis was placed on recent publications, consensus statements, guidelines, systematic reviews, meta-analyses, and relevant clinical and experimental studies. Reference lists of key publications were also screened to identify additional relevant studies. The literature search approach is illustrated in Figure 2.

## 2. Epidemiology

Pediatric MASLD is now recognized as the leading cause of chronic liver disease among children in high-income countries. Its prevalence has increased alongside the global rise in childhood obesity. However, reported prevalence rates vary considerably because they are influenced by differences in the populations studied, the diagnostic methods employed, and whether the disease is classified using the previous NAFLD definition or the newer MASLD criteria.

A 2026 systematic review and meta-analysis published in BMC Medicine, which included 56 studies involving individuals aged 5–24 years, estimated the global prevalence of pediatric metabolic dysfunction-associated steatotic liver disease (MASLD) at 7.0% (95% CI: 4.1–11.7%). The review also reported that prevalence increases with both age and calendar year, reflecting the growing burden of metabolic disease among children and adolescents [13]. Importantly, the studies included in the review used different diagnostic approaches and, where applicable, different disease nomenclature and definitions.

Higher prevalence estimates have been reported using the previous NAFLD definition. A 2024 systematic review and meta-analysis including 62 studies estimated the pooled prevalence of NAFLD at 13% in the general pediatric population and 47% among children and adolescents with obesity, although substantial variability between studies was attributed to differences in diagnostic methods and study populations [14]. Likewise, a 2026 Journal of Hepatology review reported prevalence estimates of up to 7.5%, supporting findings from recent model-based analyses [15].

Variation in reported prevalence is influenced by both true population differences and methodological factors. Imaging-based studies generally produce lower prevalence estimates than biopsy-based investigations due to differences in diagnostic sensitivity. In addition, the former NAFLD criteria, which relied on elevated alanine aminotransferase (ALT) levels and obesity, identified more cases than the 2023 MASLD definition, which requires imaging-confirmed hepatic steatosis together with at least one cardiometabolic risk factor. Using the updated criteria, an NHANES analysis estimated the prevalence among United States adolescents to be approximately 11%, compared with 16.5% under the previous NAFLD definition [16]. These differences highlight the importance of considering the study population, diagnostic method, and disease definition when interpreting and comparing prevalence estimates.

### 2.1. Prevalence in Children with Obesity

The prevalence of metabolic dysfunction-associated steatotic liver disease (MASLD) is substantially higher among children with overweight and obesity than in the general pediatric population. A 2025 meta-analysis including 176 studies and more than 57,000 participants estimated a pooled prevalence of 41.2% (95% CI: 39.7–44.6%) in children with overweight or obesity. Regional differences were also observed, with the highest prevalence reported in North America (43.6%) and the lowest in Africa (31.1%) [17].

Studies conducted in pediatric obesity clinics have reported even higher prevalence estimates, typically ranging from 44% to 47%, reflecting the increased metabolic risk in these specialized populations [4,14,18]. Similarly, children with type 2 diabetes represent another high-risk group. A 2026 meta-analysis estimated that 36.6% of pediatric patients with type 2 diabetes had MASLD, with prevalence increasing to 55% in studies that used magnetic resonance imaging (MRI), highlighting the impact of more sensitive diagnostic techniques [19,20].

### 2.2. Data Using Transient Elastography

A 2025 NHANES study including 2588 adolescents aged 12–19 years (2017–2023 cycles) reported an age-adjusted MASLD prevalence of 21.0%, with evidence of MASLD-related liver fibrosis in approximately 9–10% of affected individuals [21].

Similarly, an analysis of NHANES 2017–2020 involving participants aged 12–29 years estimated an overall MASLD prevalence of 23.9%. Among individuals with MASLD, 11% had liver fibrosis and 3.1% had cirrhosis, indicating that a proportion of affected adolescents and young adults already exhibit advanced liver disease [22].

The higher prevalence reported in these NHANES studies compared with earlier population-based estimates may reflect differences in study design, age range, and diagnostic methodology. Specifically, these studies used transient elastography to detect hepatic steatosis and included individuals meeting the current MASLD criteria, which require the presence of at least one cardiometabolic risk factor [21,22].

### 2.3. Sex, Ethnicity, and Geographic Variation

A recent review identifies pediatric MASLD as the most common chronic liver disease in children, affecting approximately 10% of the pediatric population. The authors also highlight that the prevalence of MASLD continues to increase worldwide, with cases being recognized at progressively younger ages. Furthermore, prevalence rises with increasing age throughout childhood and adolescence, while boys consistently exhibit higher prevalence rates than girls across all pediatric age groups [23].

The prevalence of pediatric MASLD varies according to sex, ethnicity, and geographic region. Male sex is a well-established risk factor, with boys consistently demonstrating approximately 1.4–1.5 times higher prevalence than girls across population-based studies [14]. In the United States, Hispanic and non-Hispanic Asian children and adolescents have a higher risk of developing MASLD compared with other ethnic groups [21].

Marked geographic variation has also been reported. Studies from Latin America have documented some of the highest prevalence estimates, ranging from 24% to 68%, a pattern largely attributed to the high prevalence of obesity and metabolic syndrome in these populations [18]. At the global level, countries with a low Socio-Demographic Index (SDI) experience a disproportionately high incidence of MASLD relative to their obesity rates. In addition, disparities in disability-adjusted life years (DALYs) between low- and high-SDI regions have continued to widen, highlighting growing inequalities in the global burden of the disease [24].

### 2.4. Disease Severity

Pediatric MASLD is not always a benign condition and may progress to significant liver disease. A 2026 review of 26 longitudinal studies reported that hepatic steatosis developing during childhood often persists into adulthood and is associated with an increased risk of type 2 diabetes mellitus (T2DM), cardiovascular disease, and liver-related mortality later in life [25]. In addition, approximately 10% of affected children have evidence of advanced liver fibrosis at the time of diagnosis, indicating that clinically significant disease may already be present during childhood.

Histologically, pediatric MASLD differs from the adult form of the disease. Children more commonly exhibit periportal steatosis and fibrosis, whereas adults typically develop centrilobular lesions. These age-related differences in histopathological features have important implications for disease staging, prognosis, and long-term clinical monitoring [26].

## 3. Risk Factors for Development of Pediatric MASLD

### 3.1. Socioeconomic Factors

A 2024 study of U.S. adolescents demonstrated that food insecurity, lower household (HH) income, and lower educational attainment were each independently associated with MASLD. The risk was particularly elevated among adolescents experiencing both food insecurity and low income, with the combination conferring a substantially increased likelihood of disease. Specifically, “food-insecure adolescents living in low income HH had a 3-fold greater risk (odds ratio [OR] 3.25, 1.31–8.08) of having MASLD” [27]. Similarly, a biopsy-based pediatric cohort study identified an association between greater neighborhood deprivation and more advanced liver disease. Compared with patients from lower SDI areas, those from high SDI areas had significantly higher median AST levels (60 vs. 47 IU/L, *p* = 0.022), NAS scores (4 vs. 3, *p* = 0.026), and prevalence of bridging fibrosis (24% vs. 10%, *p* = 0.023) [28]. Additionally, another pediatric NAFLD study reported that food insecurity was prevalent among affected children and observed that “children experiencing food insecurity presented MASLD on average 2 years before their food secure counterparts” [29].

The relationship between global SDI and MASLD burden is complex and should not be interpreted as a straightforward association in which disadvantaged regions consistently demonstrate higher prevalence. A 2024 GBD-based analysis found that, after adjustment, MASLD prevalence was associated with established metabolic risk factors, including obesity, type 2 diabetes, and low physical activity. The study also showed that the relationship between food insecurity and MASLD prevalence varied according to SDI level: in high-SDI countries, greater food insecurity was associated with increased prevalence, whereas in low-SDI countries the observed association was reversed [30]. Regarding healthcare access, a 2025 pediatric MASLD call to action highlighted “insufficient screening and disease staging practices” as well as access-related barriers, supporting the role of healthcare limitations in disease management. However, evidence directly quantifying diagnostic delays remains limited based on the available literature reviewed [30].

### 3.2. Perinatal Factors

Maternal health, intrauterine exposures, and early postnatal conditions are important determinants of metabolic programming that may influence susceptibility to MASLD later in life. Maternal factors such as a high-fat and high-carbohydrate diet, obesity, insulin resistance, gestational diabetes, maternal undernutrition, and placental dysfunction have been associated with persistent metabolic alterations in offspring. These effects may be mediated through multiple mechanisms, including mitochondrial dysfunction, epigenetic modifications, immune dysregulation, gut microbiome alterations (dysbiosis), and metabolic reprogramming of offspring stem cells. Such early-life disturbances may contribute to increased adiposity, chronic inflammation, fibrosis, and oxidative stress, while disrupting energy homeostasis and increasing the risk of MASLD development [23].

In pediatric populations, maternal metabolic status and early-life growth patterns appear to be key contributors to MASLD risk. Evidence is most consistent for associations with maternal pre-pregnancy obesity, gestational diabetes, and excessive or rapid infant weight gain. A systematic review found that maternal overweight or obesity before pregnancy was associated with an increased risk of pediatric NAFLD, whereas breastfeeding was associated with a lower risk of NAFLD, steatohepatitis, and fibrosis. However, the impact of pre-gestational diabetes remains unclear, and evidence linking preterm birth or small-for-gestational-age status with NAFLD is currently inconclusive, although rapid catch-up growth may represent an important risk factor [31]. Additional studies have highlighted the potential influence of maternal diet, smoking, and other prenatal and early postnatal exposures on childhood metabolic risk. Overall, pediatric MASLD is likely the result of a complex interplay between early metabolic programming and subsequent environmental influences, with further research required to clarify causal pathways and distinguish direct effects from shared familial metabolic risk factors [23,32].

### 3.3. Genetics

Several genes involved in hepatic lipid metabolism, triglyceride synthesis and secretion, glucose metabolism, phospholipid remodeling, and lipid droplet turnover have been implicated in the pathogenesis of MASLD. Genetic variation in these pathways influences hepatic fat accumulation, inflammation, and fibrosis, thereby modifying disease susceptibility and clinical severity. Among the identified genetic factors, the *PNPLA3* I148M variant is the most consistently validated genetic determinant and has been strongly associated with both increased disease susceptibility and greater severity of liver injury. Additional variants, including *TM6SF2* and *GCKR*, also contribute to MASLD risk, whereas *MBOAT7* and *HSD17B13* may further modify individual susceptibility and disease progression [23,33,34,35].

The impact of genetic variation is shaped by environmental and developmental factors rather than acting in isolation. Early-life exposures, including maternal obesity, gestational diabetes, and nutritional influences during pregnancy and infancy, may interact with genetic variants such as *PNPLA3* and *TM6SF2* to increase the likelihood of MASLD. Pediatric data indicate that children with obesity who also carry risk alleles are more prone to hepatic steatosis, increased ALT levels, and features associated with disease progression, including fibrosis. Studies in children have consistently identified *PNPLA3* rs738409 as a major risk variant, while additional pediatric research has linked *TM6SF2* variants with higher MASLD risk and suggested possible protective associations for *GCKR* and selected *LEPR* variants [34,35].

Current evidence therefore supports a multifactorial model in which MASLD results from the interaction of multiple genetic variants with metabolic and environmental exposures. Although *PNPLA3* represents the strongest genetic determinant identified in pediatric populations, further large-scale genomic studies are required to clarify the magnitude of these associations and their applicability across diverse ethnic groups [23,36]. However, the current evidence does not yet support the routine clinical use of these genetic variants for diagnosis or risk stratification in children with MASLD.

### 3.4. Birth Weight

Birth weight may influence pediatric MASLD risk, although the direction of the association appears complex. In a biopsy-confirmed pediatric NAFLD cohort, both low and high birth weights were more frequently observed; high birth weight was associated with greater steatosis severity and NASH, whereas low birth weight was linked to advanced fibrosis. Moreover, a more recent NHANES adolescent analysis identified an association between MASLD and high birth weight.

Low birth weight has also supporting evidence from causal analyses [37,38]. A 2024 Mendelian randomization study suggested a causal relationship between birth weight, adult BMI, and MASLD risk, indicating that low birth weight may represent a biologically plausible independent marker of later metabolic susceptibility [39].

Therefore, the association between MASLD and birthweight is characterized by a U-shaped correlation rather than a simple linear positive or negative correlation.

Evidence also suggests that rapid early postnatal growth may later increase MASLD risk. In a cohort of 162 individuals born preterm, accelerated weight gain during the first three months after term age was associated with higher Fatty Liver Index (FLI) values in early adulthood. Furthermore, 7.3% of participants with accelerated catch-up growth had a high FLI at 21 years of age, whereas none of those without accelerated catch-up growth reached this threshold, supporting a potential link between rapid early weight gain and later hepatic steatosis [40].

### 3.5. Diet and Lifestyle Factors

Diet is an important modifiable factor in the development and management of MASLD in children. Dietary habits influence energy balance, adiposity, insulin resistance, lipid metabolism, and hepatic fat accumulation. In children and adolescents, diets characterized by frequent consumption of energy-dense foods, added sugars, sugar-sweetened beverages, and ultra-processed foods are associated with an increased risk of overweight and obesity, which are major risk factors for MASLD [41]. High consumption of ultra-processed foods has also been associated with metabolic dysfunction through multiple pathways, further supporting the importance of dietary quality in MASLD prevention and management [42]. Conversely, improving overall dietary quality and adopting a balanced dietary pattern are considered important components of lifestyle management in pediatric MASLD [43].

Particular concern relates to the consumption of added sugars, especially fructose-containing foods and sugar-sweetened beverages. Excessive fructose intake may increase hepatic de novo lipogenesis and promote hepatic lipid accumulation, while also contributing to insulin resistance and other metabolic abnormalities. Recent evidence has specifically highlighted the potential role of high fructose exposure in hepatic steatosis in children, including children without obesity [44]. In addition, systematic evidence has associated sugar-sweetened beverage consumption with an increased risk of non-alcoholic fatty liver disease [45]. Therefore, limiting sugar-sweetened beverages and foods containing added sugars, particularly those providing substantial amounts of fructose, should be an important component of dietary counseling for children with or at risk of MASLD [43,44,45].

The overall dietary pattern may also be relevant to hepatic health. The Mediterranean diet, characterized by a high consumption of vegetables, fruits, legumes, whole grains, nuts, and olive oil, together with moderate consumption of fish and limited intake of highly processed foods and saturated fats, has received increasing attention in pediatric MASLD. A recent systematic review and meta-analysis of children and adolescents with MASLD reported that Mediterranean diet interventions were associated with improvements in liver enzymes, although the number of available studies was limited and their methodological heterogeneity prevents definitive conclusions regarding the superiority of this dietary pattern over other healthy diets [46].

Physical activity represents another important modifiable component of pediatric MASLD management. Regular exercise may improve insulin sensitivity, body composition, and cardiometabolic health and may contribute to reductions in hepatic fat accumulation. Conversely, sedentary behavior and insufficient physical activity may contribute to obesity and metabolic dysfunction, thereby increasing the risk of hepatic steatosis [43]. A recent systematic review of exercise interventions in pediatric MASLD found evidence of beneficial effects on hepatic and metabolic outcomes, although the available studies were heterogeneous and the overall evidence remains limited [47]. Consequently, current evidence supports promoting regular physical activity and reducing sedentary time as integral components of lifestyle intervention rather than prescribing a single specific exercise modality [43,48].

Lifestyle modification combining a healthy dietary pattern with regular physical activity remains the cornerstone of pediatric MASLD management. Available evidence suggests that lifestyle interventions can improve liver enzymes, hepatic steatosis, body composition, and metabolic risk factors in children and adolescents with MASLD [43,46,48]. Management should therefore prioritize sustainable improvements in diet quality, reduction of added sugars and sugar-sweetened beverages, increased physical activity, and reduction of sedentary behavior. Importantly, interventions should be individualized according to the child’s age, developmental stage, nutritional requirements, cultural and family context, and metabolic risk profile, while ensuring adequate growth and nutritional status [43,47]. Further well-designed pediatric trials are needed to establish the optimal dietary pattern, exercise intensity, duration, and combination of lifestyle interventions for the treatment of MASLD.

### 3.6. Microbiome

Gut microbiome dysbiosis and impaired intestinal barrier function are recognized as key contributors to the development and progression of MASLD, with microbial metabolites, including ethanol, bile acids, short-chain fatty acids, and other gut-derived products, playing important mechanistic roles [49]. Clinical evidence in pediatric MASLD is growing. A 2025 meta-analysis demonstrated that gut microbial composition changes progressively as children progress from obesity to MASLD and MASH. Compared with children with obesity and healthy controls, those with MASLD and MASH exhibited significant differences in gut microbiome diversity and composition. In particular, *Faecalibacterium prausnitzii* and *Prevotella copri* differed across disease stages, while increased *P. copri* abundance was associated with hepatic fibrosis. Fecal microbiome profiles also accurately distinguished MASLD from obesity and MASH from MASLD, highlighting their potential as non-invasive biomarkers of disease progression [50]. A recent review further emphasized the therapeutic potential of targeting the gut microbiome in pediatric MASLD through dietary and microbiome-based interventions [51].

Short-chain fatty acids (SCFAs), particularly acetate, propionate, and butyrate, are important metabolites produced by the intestinal microbiota, mainly through the fermentation of dietary fiber. Increasing evidence suggests that SCFAs may contribute to the pathophysiology of MASLD through the gut–liver axis. Following their production in the intestine, SCFAs can reach the liver through the portal circulation and influence hepatic energy metabolism, glucose and lipid homeostasis, and inflammatory pathways. They may also affect intestinal barrier integrity and host immune responses through signaling mechanisms involving G-protein-coupled receptors and other molecular pathways [51,52,53]. Alterations in gut microbial composition and SCFA production have been described in pediatric MASLD and may contribute to hepatic metabolic dysfunction and steatosis [48]. However, the relationship between individual SCFAs and MASLD is complex, and their effects may vary according to dietary intake, microbiome composition, and host metabolic status. Further pediatric studies are needed to clarify the specific role of SCFAs in disease development and progression and their potential as therapeutic targets [51,52,53].

Bile acids represent another important component of the gut–liver axis and contribute to the regulation of hepatic and systemic metabolism. Primary bile acids are synthesized in the liver and secreted into the intestine, where they undergo extensive biotransformation by the gut microbiota into secondary and other modified bile acids. These metabolites participate in enterohepatic circulation and act as signaling molecules through receptors such as the farnesoid X receptor (FXR) and Takeda G protein-coupled receptor 5 (TGR5), thereby influencing glucose and lipid metabolism, energy homeostasis, inflammation, and intestinal barrier function [51,52,53]. The interaction is bidirectional: gut microorganisms modify the bile acid pool, while bile acids exert selective effects on the composition and function of the intestinal microbiota [51,54]. Importantly, bile acid metabolism undergoes substantial changes during childhood as the gut microbiome matures, suggesting that alterations in this pathway may have particular relevance during pediatric metabolic development [54]. Emerging evidence indicates that disturbances in bile acid metabolism and signaling may contribute to metabolic dysfunction and hepatic steatosis in MASLD, although the specific role of individual bile acids and their contribution to pediatric disease progression remain incompletely understood [51,53].

These findings are derived primarily from human observational studies and demonstrate associations rather than causality.

Experimental studies provide additional mechanistic evidence. A 2018 mouse study showed that microbiota transferred from infants born to mothers with obesity increased intestinal permeability, inflammatory signaling, and susceptibility to NAFLD in offspring [55]. More recently, a 2025 mouse study demonstrated that postnatal antibiotic exposure exerted a greater effect on gut microbiome composition than prenatal exposure and was associated with increased endotoxemia and more severe hepatic steatosis [56].

### 3.7. Adiposity and Insulin Resistance

In pediatric MASLD/NAFLD, excess adiposity and insulin resistance represent the main metabolic drivers, with obesity being one of the strongest risk factors for disease development. A 2024 pediatric review highlighted the close relationship between MASLD and obesity/overweight as well as insulin resistance, while a 2026 review reported substantially higher prevalence of MASLD among children with obesity compared with the general pediatric population (47% vs. 13%) [23,57].

Insulin resistance (IR) is a key metabolic driver of MASLD, independent of *PNPLA3* genotype. Evidence from clinical studies demonstrates that individuals with MASLD have greater whole-body and adipose tissue insulin resistance compared with those without MASLD. Importantly, among individuals with similar levels of hepatic fat accumulation, IR severity is comparable between *PNPLA3 CC* and *CG/GG* genotypes, suggesting that IR contributes to MASLD development regardless of genetic background [58].

Adipose tissue insulin resistance is closely associated with visceral adiposity and central obesity. Adipose tissue insulin resistance (Adipo-IR) has been shown to positively correlate with visceral adipose tissue mass and waist circumference in children and adolescents with obesity, but not with total body fat percentage. Elevated Adipo-IR is also observed in youth with mild steatosis, suggesting that metabolic dysfunction may begin early in life and contribute to MASLD progression [59].

### 3.8. Liver Zonation

Pediatric MASLD displays a distinct hepatic zonation pattern characterized by predominant periportal (zone 1) steatosis, inflammation, and fibrosis, in contrast to the centrilobular (zone 3) pattern typically observed in adults. Multiple studies consistently support this difference, although the underlying mechanisms remain incompletely understood. Pediatric cases are associated with greater periportal involvement, whereas adult MASLD more commonly exhibits centrilobular changes [23,27].

Portal-dominant MASLD is clinically relevant because it is associated with earlier progression to advanced fibrosis [15,60]. The mechanisms underlying this pattern remain incompletely understood but likely involve developmental, immunologic, and genetic factors, including variants in *PNPLA3* and *HSD17B13* [15].

Experimental evidence suggests that early-life metabolic factors, including maternal obesity, may contribute to altered hepatic zonation. Maternal obesity has been shown to disrupt normal lobular organization in the offspring’s liver, with increased periportal region representation, reduced mid-lobular areas, and impaired zonal metabolite distribution patterns [61]. Emerging spatial omics approaches may provide further insight into these zonal alterations; however, current evidence remains largely descriptive, and the molecular mechanisms driving age-related differences in MASLD zonation require further investigation [62].

### 3.9. Environmental Factors

Childhood MASLD appears to result from a combination of early-life metabolic programming and social–environmental factors rather than a single isolated exposure. Pediatric studies identify maternal obesity, gestational diabetes, suboptimal prenatal nutrition, early nutritional patterns, breastfeeding or formula feeding differences, rapid infant weight gain, and diets high in added sugars, saturated fats, and ultra-processed foods as potential contributors to MASLD risk. These biological factors interact with broader environmental influences, including limited access to healthy foods, reduced physical activity opportunities, and unhealthy lifestyle patterns [23,63].

The association with socioeconomic conditions is particularly evident at the neighborhood level. Children with MASLD have been reported to live in areas with greater socioeconomic and environmental disadvantage compared with children with obesity without MASLD [64]. However, findings regarding the relationship between deprivation and disease severity remain inconsistent, with some studies showing no association between area-level deprivation and liver injury markers or histological activity. Overall, current evidence is largely based on reviews and observational cohorts, highlighting the need for further studies to distinguish the independent contributions of prenatal, household, and neighborhood-level factors [65].

### 3.10. Other Factors

Disease risk is further influenced by additional factors beyond adiposity and insulin resistance. A cohort study of children with obesity found that NASH was associated with higher BMI-SDS, sex, hyperuricemia, and insulin resistance [64]. Therefore, while obesity and insulin resistance form the central pathogenic framework, factors such as dyslipidemia, sex, uric acid levels, genetic susceptibility, and early-life exposures may further modify disease risk and severity [23].

**Figure 3:**
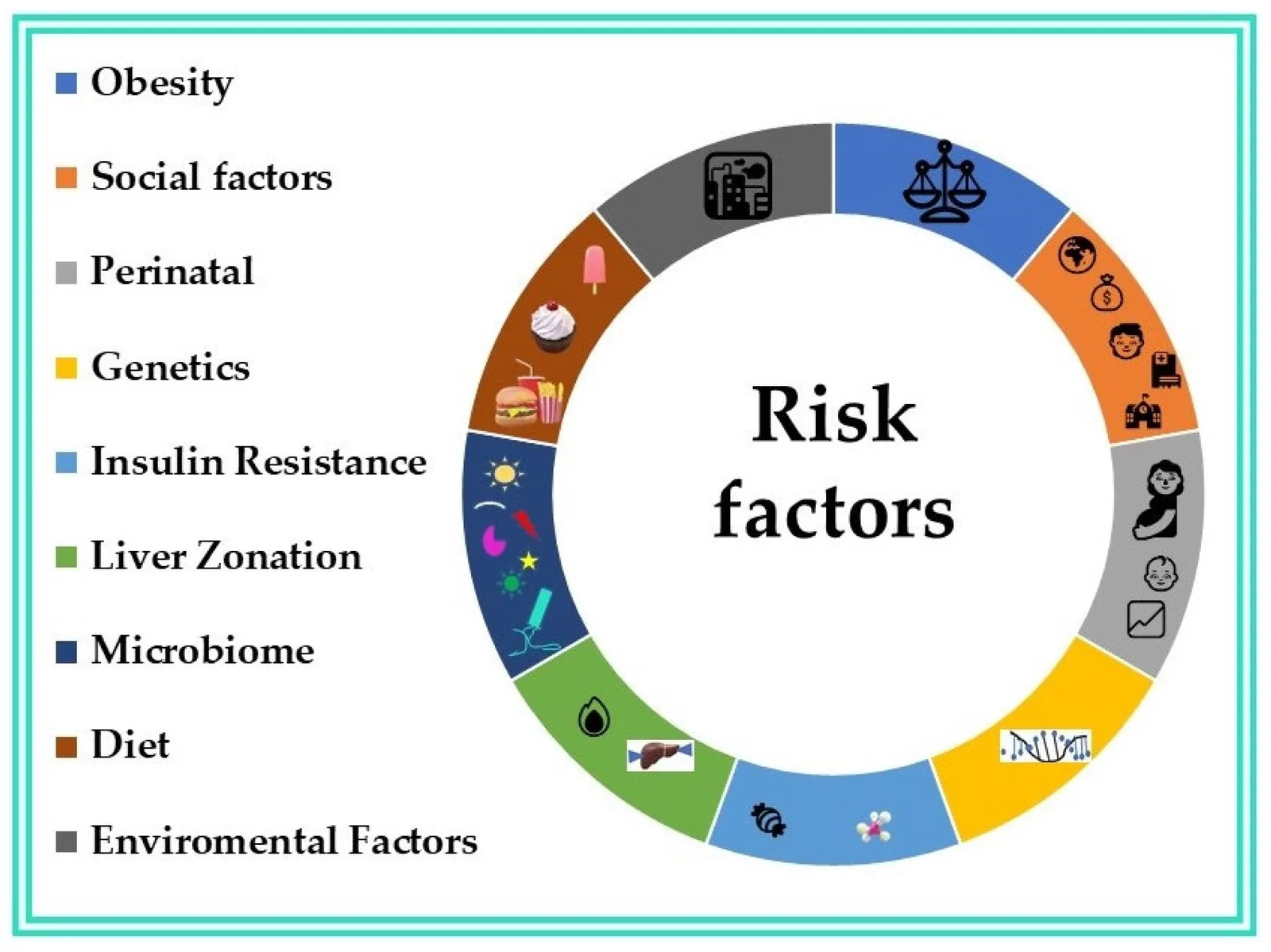
Risk factors contributing to the development of pediatric metabolic dysfunction-associated steatotic liver disease (MASLD). The pathogenesis of pediatric MASLD is multifactorial and involves the complex interplay of genetic, perinatal, metabolic, environmental, socioeconomic, and microbial factors. Key contributors include altered birth weight and perinatal exposures, genetic susceptibility, adiposity and insulin resistance, gut microbiome alterations, liver zonation, and environmental and socioeconomic influences. These factors may interact throughout early life and childhood to promote hepatic steatosis and increase susceptibility to MASLD.

Main risk factors for the development of MASLD are illustrated in Figure 3.

## 4. Lean MASLD

Lean MASLD is increasingly recognized as a clinically important phenotype occurring in individuals without obesity. Although most evidence derives from adult populations, lean MASLD has been associated with insulin resistance, increased visceral adiposity, and adverse liver outcomes despite a more favorable metabolic profile than obesity-associated disease [66]. It also presents unique diagnostic challenges and has been associated with mortality rates comparable to or higher than those observed in non-lean MASLD [67,68].

In contrast to adult populations, lean pediatric MASLD has not yet been well characterized as a distinct clinical entity. While pediatric MASLD affects approximately 10% of children overall, with prevalence increasing to nearly 47% among children with obesity [69], the prevalence, clinical characteristics, natural history, and long-term outcomes of MASLD in lean children remain largely undefined. Although evidence remains limited, available studies suggest that genetic susceptibility may play role in lean pediatric MASLD. Polymorphisms in *ENPP1* and *IRS1* have been implicated in disease susceptibility, whereas variants in *PNPLA3* and *TM6SF2* have been associated with greater hepatic steatosis severity. Environmental factors, particularly excessive fructose consumption, may also contribute to disease development in this subgroup [66]. However, studies specifically examining lean pediatric MASLD are scarce, representing an important gap in the current literature. Further research is needed to better define its epidemiology, pathophysiology, clinical characteristics, and prognosis, as well as to inform appropriate screening, diagnosis, and management strategies for this potentially underrecognized subgroup.

Potential causes of liver steatosis in apparently asymptomatic children and adolescents, regardless of body weight status, are summarized in Table 1 (modified from reference [70]).

## 5. Extrahepatic Comorbidities

Pediatric MASLD is frequently accompanied by a broad spectrum of extrahepatic manifestations, with cardiometabolic abnormalities representing the most consistently reported comorbidities [71,72,73]. The prevalence of MASLD among children with established metabolic disorders, including diabetes mellitus, dyslipidemia, and hypertension, remains incompletely defined, largely because routine screening for hepatic steatosis is not universally implemented.

### 5.1. Cardiovascular Complications

Pediatric MASLD is increasingly recognized as a marker of long-term cardiovascular risk. Although cardiovascular events are uncommon during childhood, longitudinal studies indicate that MASLD is associated with early vascular and cardiac abnormalities, including subclinical vascular remodeling, increased arterial stiffness, hypertension, and altered cardiac structure and function. Evidence from the Generation R prospective birth cohort, which included more than 3000 liver MRI assessments in 10-year-old children, demonstrated that increased liver fat content was associated with adverse cardiometabolic profiles, including higher blood pressure, insulin resistance, and unfavorable serum lipid levels [72]. These associations persisted after adjustment for BMI and visceral fat mass, suggesting that hepatic steatosis may contribute independently to cardiovascular risk. Notably, even modest elevations in liver fat, below the threshold of overt steatosis, were associated with increased odds of cardiometabolic abnormalities, with stronger associations observed among children with overweight or obesity [72]. In children and young adults with biopsy-confirmed NAFLD, an increased risk of major adverse cardiovascular events has been reported compared with controls (adjusted HR 2.33), with a higher risk among those with steatohepatitis (HR 5.27) [74]. Recent pediatric longitudinal studies have demonstrated associations between childhood MASLD and adverse subclinical cardiovascular measures, suggesting early vascular impairment [75]. Furthermore, improvement of MASLD has been associated with healthier cardiovascular structural parameters, supporting the potential reversibility of early cardiovascular changes through metabolic intervention [76]. These findings emphasize that MASLD represents a systemic cardiometabolic disorder and support early cardiovascular risk assessment and management in affected children.

### 5.2. Metabolic Complications

Insulin resistance (IR) is a central mechanism in the pathogenesis of pediatric MASLD and contributes to disease development independently of PNPLA3 genetic variation [58]. Adolescence represents a particularly vulnerable period, as a physiological reduction in insulin sensitivity of approximately 25–50% occurs during puberty, independent of body mass index (BMI) and adiposity, before returning to prepubertal levels after maturation, potentially facilitating MASLD development [15,77]. In children with severe obesity, adipose tissue IR is strongly associated with hepatic fat accumulation, particularly in the presence of visceral adiposity and increased waist circumference rather than total body fat [61]. The relationship between MASLD and type 2 diabetes mellitus (T2DM) is bidirectional, with dysglycemia accelerating liver disease progression and MASLD increasing the risk of overt T2DM. Children with NAFLD and prediabetes or newly diagnosed T2DM are more likely to develop steatohepatitis and progressive fibrosis [78], while longitudinal studies have demonstrated a substantially increased incidence of youth-onset T2DM among children with MASLD, especially those with severe obesity and advanced histological disease [79,80]. Importantly, even modest weight reduction through obesity treatment significantly lowers the risk of T2DM, highlighting the benefits of early metabolic intervention [80]. A smaller continuous Glucose Monitoring (CGM) study in prepubertal children better revealed glycemic dysregulation that fasting glucose and HbA1c could miss, and older OGTT data in obese children with fatty liver showed more impaired glucose tolerance or T2D than in obese peers without it [69]. These findings emphasize that pediatric MASLD is a high-risk metabolic disorder requiring early identification and management of insulin resistance and glucose abnormalities.

### 5.3. Obstructive Sleep Apnea (OSA)

OSA is a common extrahepatic comorbidity in pediatric MASLD, with reported prevalence rates of up to 70% [81,82]. OSA has been independently associated with more severe steatohepatitis and advanced liver fibrosis, even after adjustment for BMI and central adiposity [81,82]. A meta-analysis by Chen et al. involving 1133 children and adolescents confirmed significant associations between OSA and elevated aminotransferase levels as well as liver fibrosis, although no significant association with hepatic inflammation was observed [83]. Intermittent hypoxia and oxidative stress are considered key mechanisms underlying OSA-related liver injury, with oxidative stress markers correlating with NASH severity [84]. Importantly, treatment with continuous positive airway pressure (CPAP) has been associated with improvements in liver enzyme levels and cardiometabolic parameters, suggesting that early recognition and management of OSA may improve outcomes in children with MASLD [85].

### 5.4. Bone Health

Emerging evidence indicates that children with MASLD may have impaired skeletal health, with lower whole-body and lumbar spine bone mineral density (BMD) z-scores compared with unaffected peers, even after adjustment for age, BMI, and race/ethnicity [71,86]. Chronic low-grade inflammation may contribute to reduced bone accrual, as higher high-sensitivity C-reactive protein (hs-CRP) levels have been inversely associated with BMD measures [86]. Vitamin D deficiency is also frequently reported in children with MASLD; however, its association with liver disease severity remains unclear [87]. Recent reviews suggest that metabolic abnormalities, inflammation, insulin resistance, and altered adipokine signaling may contribute to the relationship between MASLD and reduced bone quality [88]. Although current evidence supports an association between pediatric MASLD and lower BMD, further longitudinal studies are required to clarify mechanisms and determine the long-term impact on fracture risk.

### 5.5. Renal Complications

Kidney injury is increasingly recognized as an important cardiometabolic comorbidity associated with pediatric MASLD. Recent large pediatric cohorts have demonstrated that renal abnormalities are relatively common among children with MASLD, particularly in those with more advanced liver fibrosis [89,90]. In a biopsy-confirmed pediatric NAFLD cohort, approximately one-third of children showed abnormal renal function, including glomerular hyperfiltration and reduced estimated glomerular filtration rate (eGFR), with hyperfiltration independently associated with greater liver disease activity [91]. Although these findings suggest a link between hepatic and renal involvement in pediatric MASLD, the long-term progression of kidney injury, its relationship with liver disease evolution, and the potential independent contribution of genetic factors such as the PNPLA3 I148M variant remain unclear [89,90].

### 5.6. Psychosocial Consequences

MASLD is increasingly recognized as a condition with important psychosocial consequences. Available evidence suggests that affected children frequently experience reduced health-related quality of life, fatigue, emotional distress, anxiety, depressive symptoms, and other neuropsychiatric manifestations. Current data also indicate that the severity of psychosocial impairment does not necessarily correlate with the degree of liver damage, suggesting that psychosocial burden may develop independently of histological disease severity. Furthermore, children with neurodevelopmental or intellectual disabilities may be at increased risk of hepatic steatosis, supporting a bidirectional relationship between MASLD and psychosocial health. However, pediatric evidence remains limited, and further longitudinal studies are needed to clarify underlying mechanisms and determine whether treatment of MASLD improves psychological well-being and quality of life [92,93].

### 5.7. Long-Term Outcomes

Recent longitudinal studies demonstrate that pediatric MASLD is associated with increased premature mortality and substantial long-term morbidity. A nationwide Swedish cohort of 718 individuals with biopsy-confirmed MASLD diagnosed before 25 years of age reported significantly higher all-cause mortality than in matched population controls over a median follow-up of approximately 16 years, with liver disease, cancer, and cardiometabolic disorders accounting for most excess deaths [11]. These findings are supported by the LIVERS cohort, which followed 1096 children with MASLD for a mean of 8.5 years and reported a mortality rate of 398 per 100,000 person-years, with nearly half of deaths being liver-related. The cumulative incidence of cirrhosis was 4.7%, while dyslipidemia, hypertension, obstructive sleep apnea, and type 2 diabetes were the most common extrahepatic comorbidities. Male sex and lower HDL cholesterol independently predicted mortality [12]. Collectively, these studies highlight pediatric MASLD as a progressive multisystem disease requiring early diagnosis, comprehensive risk factor assessment, and long-term multidisciplinary follow-up.

## 6. Diagnosis and Screening

Diagnosis of pediatric MASLD currently relies on biochemical screening with ALT, supported by imaging techniques and, when required, histological assessment. Current guidelines recommend ALT ≥ 30 IU/L as an initial screening threshold in children older than 10 years with obesity [94]. ALT is a screening marker and should not be considered diagnostic of MASLD in isolation. Liver biopsy remains the reference standard for confirming diagnosis, assessing steatohepatitis and fibrosis severity, and resolving diagnostic uncertainty; however, its invasive nature limits widespread clinical application [95]. Several non-invasive biomarkers or their combination are being investigated, including a six-protein inflammatory panel consisting of FGF-21, CDCP1, CD244, OPG, Flt3L, and MCP-1, which demonstrated promising diagnostic performance with an area under the curve (AUC) of 0.84 in a pediatric cohort [96]. Despite advances in diagnostic strategies, MASLD remains substantially underrecognized, with real-world data from 908 children showing that only 7.4% had a clinical diagnosis despite 14.5% meeting diagnostic criteria retrospectively [47,97]. These findings highlight the need for improved screening pathways and validated non-invasive tools for early detection of pediatric MASLD.

A personalized approach to pediatric MASLD diagnosis should be tailored to the child’s age, degree of metabolic risk, clinical presentation, and likelihood of progressive liver disease. Initial assessment includes a detailed evaluation of obesity pattern, family history, diet and lifestyle factors, insulin resistance, dyslipidemia, hypertension, and associated comorbidities, combined with ALT measurement using pediatric reference limits. Children with persistent ALT elevation or high-risk features require individualized evaluation to confirm hepatic steatosis, exclude alternative causes of liver injury, and assess fibrosis risk. MRI-based liver fat quantification may be considered when confirmation or quantification of hepatic steatosis is needed, particularly when ultrasound findings are inconclusive, while elastography may be used to assess fibrosis risk in children with persistent ALT elevation or other features suggesting progressive disease. Liver biopsy should be reserved for children with diagnostic uncertainty, suspected MASH or advanced fibrosis, or when histological assessment is required to guide management. This risk-stratified strategy aims to avoid unnecessary investigations in low-risk children while ensuring early identification and management of those at increased risk for progressive MASLD [1,3,15,72,94].

## 7. Personalized Management and Risk Stratification

Management of pediatric MASLD should be individualized according to each child’s metabolic profile, severity of liver involvement, risk of fibrosis progression, and psychosocial context. Initial assessment should integrate clinical history, growth pattern, obesity severity, family history, metabolic comorbidities, ALT trajectory, and non-invasive tests (NITs) to identify children who require closer monitoring or specialist referral [47,72,98].

Children with low-risk features may benefit from a personalized lifestyle program delivered by the primary pediatric team, focusing on age-appropriate nutritional strategies, increased physical activity, behavioral support, and family-based interventions [15].

Those with higher-risk characteristics, including persistent or markedly elevated ALT, youth-onset type 2 diabetes, abnormal fibrosis markers, or evidence of disease progression, should receive coordinated multidisciplinary care involving pediatric hepatology, nutrition, endocrinology, physical activity, and behavioral health specialists. Because no single NIT accurately captures the full spectrum of disease severity in children, clinical decisions should rely on the integration of multiple risk indicators and their evolution over time. When findings are discordant or suggest advanced disease, further evaluation, including liver biopsy in selected cases, may be considered [72,94,99].

Therapeutic strategies should be adapted to the child’s needs, preferences, family circumstances, and response to intervention. Lifestyle modification remains the cornerstone of treatment, while anti-obesity pharmacotherapy or metabolic and bariatric surgery may be considered for selected adolescents with severe obesity and persistent disease despite optimized lifestyle management.

Continuous follow-up with dynamic reassessment allows timely escalation or de-escalation of care and supports a precision-based approach to reducing long-term hepatic and cardiometabolic complications of pediatric MASLD.

Future progress in pediatric MASLD will require coordinated efforts to improve early identification, non-invasive risk stratification, lifestyle interventions, therapeutic options, and understanding of disease heterogeneity. The following priorities may help guide future research and clinical practice (Table 2).

## 8. Conclusions

MASLD in children represents an increasingly important global health challenge, driven by the rising prevalence of obesity, insulin resistance, and associated metabolic risk factors during childhood. It should not be viewed as an isolated liver disease, but rather as the hepatic manifestation of a broader multisystem metabolic disorder associated with increased cardiometabolic risk. Therefore, its management requires a holistic and multidisciplinary approach involving paediatric hepatologists, endocrinologists, obesity management teams, general paediatricians, psychologists, dietitians, and active family participation. This integrated strategy is essential for addressing underlying risk factors, improving metabolic health, and preventing long-term disease progression.

The complexity of paediatric MASLD, influenced by genetic susceptibility, environmental exposures, lifestyle behaviours, and individual metabolic profiles, highlights the limitations of a one-size-fits-all approach to prevention and treatment. A personalised medicine approach provides an opportunity to enhance early identification, risk stratification, and management by integrating clinical characteristics, genetic information, biomarkers, microbiome profiles, and lifestyle factors. Early recognition of children at higher risk of disease progression may allow targeted interventions before irreversible liver damage develops. Furthermore, personalised lifestyle interventions and emerging pharmacological therapies tailored to specific disease mechanisms may improve treatment effectiveness and long-term outcomes. However, the integration of genomic, microbiome, and other multi-omics approaches into routine clinical practice remains largely investigational and requires further validation before widespread clinical implementation.

## Figures and Tables

**Table 1:** Potential causes of liver steatosis in apparently asymptomatic children and adolescents.

Category	Examples/Causes of Liver Steatosis in Children	Clues Suggesting Diagnosis
Metabolic dysfunction-associated steatotic liver disease (MASLD)	Obesity, insulin resistance, metabolic syndrome	Central obesity, dyslipidemia, elevated ALT, family history of metabolic disease
Genetic and metabolic disorders	Fatty acid oxidation defects, mitochondrial disorders, lysosomal acid lipase deficiency, Wilson disease, glycogen storage diseases, abetalipoproteinemia	Early age of onset, failure to thrive, hypoglycemia, neurologic findings, atypical presentation
Syndromes	Bardet–Biedl syndrome, Cohen syndrome, 1p36 deletion syndrome, Dorfman–Chanarin, Prader–Willi syndrome, polycystic ovary syndrome (PCOS),	Special phenotypes
Medication-induced steatosis	Corticosteroids, valproic acid, methotrexate, antiretroviral drugs, chemotherapy agents	History of medication exposure, temporal association
Nutritional causes	Malnutrition, rapid weight loss, eating disorders, total parenteral nutrition (TPN)	Weight loss, inadequate nutrition, chronic illness, prolonged TPN
Endocrine disorders	Hypothyroidism, growth hormone deficiency, Cushing syndrome	Growth abnormalities, hormonal symptoms, abnormal endocrine tests
Infectious causes	Hepatitis C, HIV-associated liver disease	Positive serology, risk factors, chronic infection
Autoimmune/inflammatory liver disease	Autoimmune hepatitis overlap syndromes, inflammatory bowel disease-associated liver disease	Elevated autoimmune markers, increased inflammatory activity
Alcohol-related liver disease	Alcohol exposure in adolescents	History of alcohol use, compatible clinical findings

**Table 2:** Future research priorities and clinical recommendations for pediatric MASLD.

Area	Future Perspectives and Recommendations
**Early detection and screening**	Improve early identification of children at risk of MASLD using accessible and non-invasive approaches.
**Risk stratification**	Identify reliable clinical, biochemical, imaging, genetic, and molecular markers for predicting disease severity and fibrosis progression.
**Longitudinal studies**	Conduct prospective pediatric studies to better characterize disease progression, regression, and long-term outcomes.
**Lifestyle intervention**	Strengthen evidence for sustainable, family-centered dietary, physical activity, and behavioral interventions.
**Gut–liver axis**	Further investigate the contribution of the gut microbiome and its metabolites to MASLD pathogenesis and as therapeutic target
**Short-chain fatty acids**	Clarify the role of acetate, propionate, and butyrate in hepatic glucose and lipid metabolism and their potential relevance as therapeutic or prognostic targets.
**Bile acids**	Investigate alterations in bile acid metabolism and microbiome–bile acid interactions, including signaling through FXR and TGR5, in pediatric MASLD.
**Non-invasive biomarkers**	Develop and validate biomarkers capable of assessing steatosis, steatohepatitis, and fibrosis while reducing the need for liver biopsy.
**Therapeutic strategies**	Evaluate pharmacological and microbiome-targeted interventions specifically in pediatric populations, with particular attention to efficacy and long-term safety.
**Multidisciplinary management**	Promote collaboration among pediatricians, hepatologists, nutrition specialists, psychologists, and other healthcare professionals.
**Personalized medicine**	Investigate genetic, metabolic, environmental, and microbiome-related factors that may contribute to individual differences in disease susceptibility and treatment response.
**Transition to adulthood**	Establish structured long-term follow-up and transition programs to address the potential hepatic and cardiometabolic consequences of pediatric MASLD.
**Research priorities**	Encourage multicenter studies using standardized definitions, diagnostic approaches, and outcome measures to strengthen the pediatric evidence base.

## Data Availability

The original contributions presented in this study are included in the article. Further inquiries can be directed to the corresponding author.

## Abbreviations

The following abbreviations are used in this manuscript:

MASLD.	Metabolic dysfunction-associated steatotic liver disease
NAFLD	Non-alcoholic fatty liver disease
NASH	Non-alcoholic steatohepatitis
MASH	metabolic dysfunction-associated steatohepatitis
ALT	Alanine aminotransferase
NHANES	National Health and Nutrition Examination Survey
HH	Household
MRI	Magnetic resonance imaging
SDI	Socio-Demographic Index
DALYs	disability-adjusted life years
NAS	NAFLD Activity Score
FLI	Fatty Liver Index
SCFAs	Short-chain fatty acids
FXR	Farnesoid X receptor
TGR5	Takeda G protein-coupled receptor 5
Adipo-IR	Adipose tissue insulin resistance
IR	Insulin Resistance
CGM	Continuous Glucose Monitoring
OSA	Obstructive sleep apnea
CPAP	Continuous positive airway pressure
BMD	Bone mineral density
BMI	Body mass Index
GFR	Glomerular filtration rate
NITs	Non-invasive tests
